# Series: Practical guidance to qualitative research. Part 3: Sampling, data collection and analysis

**DOI:** 10.1080/13814788.2017.1375091

**Published:** 2017-12-04

**Authors:** Albine Moser, Irene Korstjens

**Affiliations:** ^a^ Faculty of Health Care, Research Centre Autonomy and Participation of Chronically Ill People, Zuyd University of Applied Sciences Heerlen The Netherlands; ^b^ Faculty of Health, Medicine and Life Sciences, Department of Family Medicine, Maastricht University Maastricht The Netherlands; ^c^ Faculty of Health Care, Research Centre for Midwifery Science, Zuyd University of Applied Sciences Maastricht The Netherlands

**Keywords:** General practice/family medicine, general qualitative designs and methods, sampling, data collection, analysis

## Abstract

In the course of our supervisory work over the years, we have noticed that qualitative research tends to evoke a lot of questions and worries, so-called frequently asked questions (FAQs). This series of four articles intends to provide novice researchers with practical guidance for conducting high-quality qualitative research in primary care. By ‘novice’ we mean Master’s students and junior researchers, as well as experienced quantitative researchers who are engaging in qualitative research for the first time. This series addresses their questions and provides researchers, readers, reviewers and editors with references to criteria and tools for judging the quality of qualitative research papers. The second article focused on context, research questions and designs, and referred to publications for further reading. This third article addresses FAQs about sampling, data collection and analysis. The data collection plan needs to be broadly defined and open at first, and become flexible during data collection. Sampling strategies should be chosen in such a way that they yield rich information and are consistent with the methodological approach used. Data saturation determines sample size and will be different for each study. The most commonly used data collection methods are participant observation, face-to-face in-depth interviews and focus group discussions. Analyses in ethnographic, phenomenological, grounded theory, and content analysis studies yield different narrative findings: a detailed description of a culture, the essence of the lived experience, a theory, and a descriptive summary, respectively. The fourth and final article will focus on trustworthiness and publishing qualitative research.

Key points on sampling, data collection and analysis

- The data collection plan needs to be broadly defined and open during data collection.
- Sampling strategies should be chosen in such a way that they yield rich information and are consistent with the methodological approach used.
- Data saturation determines sample size and is different for each study.
- The most commonly used data collection methods are participant observation, face-to-face in-depth interviews and focus group discussions.
- Analyses of ethnographic, phenomenological, grounded theory, and content analysis studies yield different narrative findings: a detailed description of a culture, the essence of the lived experience, a theory or a descriptive summary, respectively.

## Introduction

This article is the third paper in a series of four articles aiming to provide practical guidance to qualitative research. In an introductory paper, we have described the objective, nature and outline of the Series [1]. Part 2 of the series focused on context, research questions and design of qualitative research [2]. In this paper, Part 3, we address frequently asked questions (FAQs) about sampling, data collection and analysis.

## Sampling

### What is a sampling plan?

A sampling plan is a formal plan specifying a sampling method, a sample size, and procedure for recruiting participants (Box 1) [3]. A qualitative sampling plan describes how many observations, interviews, focus-group discussions or cases are needed to ensure that the findings will contribute rich data. In *quantitative* studies, the sampling plan, including sample size, is determined in detail in beforehand but *qualitative* research projects start with a broadly defined sampling plan. This plan enables you to include a variety of settings and situations and a variety of participants, including negative cases or extreme cases to obtain rich data. The key features of a qualitative sampling plan are as follows. First, participants are always sampled deliberately. Second, sample size differs for each study and is small. Third, the sample will emerge during the study: based on further questions raised in the process of data collection and analysis, inclusion and exclusion criteria might be altered, or the sampling sites might be changed. Finally, the sample is determined by conceptual requirements and not primarily by representativeness. You, therefore, need to provide a description of and rationale for your choices in the sampling plan. The sampling plan is appropriate when the selected participants and settings are sufficient to provide the information needed for a full understanding of the phenomenon under study.

Box 1. Sampling strategies in qualitative research. Based on Polit & Beck [3].

**Table:** 

Sampling	Definition
Purposive sampling	Selection of participants based on the researchers’ judgement about what potential participants will be most informative.
Criterion sampling	Selection of participants who meet pre-determined criteria of importance.
Theoretical sampling	Selection of participants based on the emerging findings to ensure adequate representation of theoretical concepts.
Convenience sampling	Selection of participants who are easily available.
Snowball sampling	Selection of participants through referrals by previously selected participants or persons who have access to potential participants.
Maximum variation sampling	Selection of participants based on a wide range of variation in backgrounds.
Extreme case sampling	Purposeful selection of the most unusual cases.
Typical case sampling	Selection of the most typical or average participants.
Confirming and disconfirming sampling	Confirming and disconfirming cases sampling supports checking or challenging emerging trends or patterns in the data.

Some practicalities: a critical first step is to select settings and situations where you have access to potential participants. Subsequently, the best strategy to apply is to recruit participants who can provide the richest information. Such participants have to be knowledgeable on the phenomenon and can articulate and reflect, and are motivated to communicate at length and in depth with you. Finally, you should review the sampling plan regularly and adapt when necessary.

### What sampling strategies can I use?

Sampling is the process of selecting or searching for situations, context and/or participants who provide rich data of the phenomenon of interest [3]. In qualitative research, you sample deliberately, not at random. The most commonly used deliberate sampling strategies are purposive sampling, criterion sampling, theoretical sampling, convenience sampling and snowball sampling. Occasionally, the ‘maximum variation,’ ‘typical cases’ and ‘confirming and disconfirming’ sampling strategies are used. Key informants need to be carefully chosen. Key informants hold special and expert knowledge about the phenomenon to be studied and are willing to share information and insights with you as the researcher [3]. They also help to gain access to participants, especially when groups are studied. In addition, as researcher, you can validate your ideas and perceptions with those of the key informants.

### What is the connection between sampling types and qualitative designs?

The ‘big three’ approaches of ethnography, phenomenology, and grounded theory use different types of sampling.

In ethnography, the main strategy is purposive sampling of a variety of key informants, who are most knowledgeable about a culture and are able and willing to act as representatives in revealing and interpreting the culture. For example, an ethnographic study on the cultural influences of communication in maternity care will recruit key informants from among a variety of parents-to-be, midwives and obstetricians in midwifery care practices and hospitals.

Phenomenology uses criterion sampling, in which participants meet predefined criteria. The most prominent criterion is the participant’s experience with the phenomenon under study. The researchers look for participants who have shared an experience, but vary in characteristics and in their individual experiences. For example, a phenomenological study on the lived experiences of pregnant women with psychosocial support from primary care midwives will recruit pregnant women varying in age, parity and educational level in primary midwifery practices.

Grounded theory usually starts with purposive sampling and later uses theoretical sampling to select participants who can best contribute to the developing theory. As theory construction takes place concurrently with data collection and analyses, the theoretical sampling of new participants also occurs along with the emerging theoretical concepts. For example, one grounded theory study tested several theoretical constructs to build a theory on autonomy in diabetes patients [4]. In developing the theory, the researchers started by purposefully sampling participants with diabetes differing in age, onset of diabetes and social roles, for example, employees, housewives, and retired people. After the first analysis, researchers continued with theoretically sampling, for example, participants who differed in the treatment they received, with different degrees of care dependency, and participants who receive care from a general practitioner (GP), at a hospital or from a specialist nurse, etc.

In addition to the ‘big three’ approaches, content analysis is frequently applied in primary care research, and very often uses purposive, convenience, or snowball sampling. For instance, a study on peoples’ choice of a hospital for elective orthopaedic surgery used snowball sampling [5]. One elderly person in the private network of one researcher personally approached potential respondents in her social network by means of personal invitations (including letters). In turn, respondents were asked to pass on the invitation to other eligible candidates.

Sampling is also dependent on the characteristics of the setting, e.g., access, time, vulnerability of participants, and different types of stakeholders. The setting, where sampling is carried out, is described in detail to provide thick description of the context, thereby, enabling the reader to make a transferability judgement (see Part 3: transferability). Sampling also affects the data analysis, where you continue decision-making about whom or what situations to sample next. This is based on what you consider as still missing to get the necessary information for rich findings (see Part 1: emergent design). Another point of attention is the sampling of ‘invisible groups’ or vulnerable people. Sampling of these participants would require applying multiple sampling strategies, and more time calculated in the project planning stage for sampling and recruitment [6].

### How do sample size and data saturation interact?

A guiding principle in qualitative research is to sample only until data saturation has been achieved. Data saturation means the collection of qualitative data to the point where a sense of closure is attained because new data yield redundant information [3].

Data saturation is reached when no new analytical information arises anymore, and the study provides maximum information on the phenomenon. In quantitative research, by contrast, the sample size is determined by a power calculation. The usually small sample size in qualitative research depends on the information richness of the data, the variety of participants (or other units), the broadness of the research question and the phenomenon, the data collection method (e.g., individual or group interviews) and the type of sampling strategy. Mostly, you and your research team will jointly decide when data saturation has been reached, and hence whether the sampling can be ended and the sample size is sufficient. The most important criterion is the availability of enough in-depth data showing the patterns, categories and variety of the phenomenon under study. You review the analysis, findings, and the quality of the participant quotes you have collected, and then decide whether sampling might be ended because of data saturation. In many cases, you will choose to carry out two or three more observations or interviews or an additional focus group discussion to confirm that data saturation has been reached.

When designing a qualitative sampling plan, we (the authors) work with estimates. We estimate that ethnographic research should require 25–50 interviews and observations, including about four-to-six focus group discussions, while phenomenological studies require fewer than 10 interviews, grounded theory studies 20–30 interviews and content analysis 15–20 interviews or three-to-four focus group discussions. However, these numbers are very tentative and should be very carefully considered before using them. Furthermore, qualitative designs do not always mean small sample numbers. Bigger sample sizes might occur, for example, in content analysis, employing rapid qualitative approaches, and in large or longitudinal qualitative studies.

## Data collection

### What methods of data collection are appropriate?

The most frequently used data collection methods are participant observation, interviews, and focus group discussions. Participant observation is a method of data collection through the participation in and observation of a group or individuals over an extended period of time [3]. Interviews are another data collection method in which an interviewer asks the respondents questions [6], face-to-face, by telephone or online. The qualitative research interview seeks to describe the meanings of central themes in the life world of the participants. The main task in interviewing is to understand the meaning of what participants say [5]. Focus group discussions are a data collection method with a small group of people to discuss a given topic, usually guided by a moderator using a questioning-route [8]. It is common in qualitative research to combine more than one data collection method in one study. You should always choose your data collection method wisely. Data collection in qualitative research is unstructured and flexible. You often make decisions on data collection while engaging in fieldwork, the guiding questions being with whom, what, when, where and how. The most basic or ‘light’ version of qualitative data collection is that of open questions in surveys. Box 2 provides an overview of the ‘big three’ qualitative approaches and their most commonly used data collection methods.

Box 2. Qualitative data collection methods.

**Table:** 

	Definition	Aim	Ethno-graphy	Pheno-menology	Grounded theory	Content analysis
Participants of observations	Participation in and observation of people or groups.	To obtain a close and intimate familiarity with a given group of individuals and their practices through intensive involvement with people in their environment, usually over an extended period.	Suitable		Very rare	Sometimes
Face-to-face in-depths Interviews	A conversation where the researcher poses questions and the participants provide answers face-to-face, by telephone or via mail.	To elicit the participant’s experiences, perceptions, thoughts and feelings.	Suitable	Suitable	Suitable	Suitable
Focus group discussion	Interview with a group of participants to answer questions on a specific topic face-to-face or via mail; people who participate interact with each other.	To examine different experiences, perceptions, thoughts and feelings among various participants or parties.	Suitable		Sometimes	Suitable

### What role should I adopt when conducting participant observations?

What is important is to immerse yourself in the research setting, to enable you to study it from the inside. There are four types of researcher involvement in observations, and in your qualitative study, you may apply all four. In the first type, as ‘complete participant’, you become part of the setting and play an insider role, just as you do in your own work setting. This role might be appropriate when studying persons who are difficult to access. The second type is ‘active participation’. You have gained access to a particular setting and observed the group under study. You can move around at will and can observe in detail and depth and in different situations. The third role is ‘moderate participation’. You do not actually work in the setting you wish to study but are located there as a researcher. You might adopt this role when you are not affiliated to the care setting you wish to study. The fourth role is that of the ‘complete observer’, in which you merely observe (bystander role) and do not participate in the setting at all. However, you cannot perform any observations without access to the care setting. Such access might be easily obtained when you collect data by observations in your own primary care setting. In some cases, you might observe other care settings, which are relevant to primary care, for instance observing the discharge procedure for vulnerable elderly people from hospital to primary care.

### How do I perform observations?

It is important to decide what to focus on in each individual observation. The focus of observations is important because you can never observe everything, and you can only observe each situation once. Your focus might differ between observations. Each observation should provide you with answers regarding ‘Who do you observe?’, ‘What do you observe’, ‘Where does the observation take place?’, ‘When does it take place?’, ‘How does it happen?’, and ‘Why does it happen as it happens?’ Observations are not static but proceed in three stages: descriptive, focused, and selective. *Descriptive* means that you observe, on the basis of general questions, everything that goes on in the setting. *Focused* observation means that you observe certain situations for some time, with some areas becoming more prominent. *Selective* means that you observe highly specific issues only. For example, if you want to observe the discharge procedure for vulnerable elderly people from hospitals to general practice, you might begin with broad observations to get to know the general procedure. This might involve observing several different patient situations. You might find that the involvement of primary care nurses deserves special attention, so you might then focus on the roles of hospital staff and primary care nurses, and their interactions. Finally, you might want to observe only the specific situations where hospital staff and primary care nurses exchange information. You take field notes from all these observations and add your own reflections on the situations you observed. You jot down words, whole sentences or parts of situations, and your reflections on a piece of paper. After the observations, the field notes need to be worked out and transcribed immediately to be able to include detailed descriptions.

Box 3. Further reading on interviews and focus group discussion.

**Table:** 

Face-to-face interviews	Brinkmann S, Kvale S. Interviews. Learning the craft of qualitative research interviewing. 3rd ed. Sage: London; 2014.Rubin HJ, Rubin IS. Qualitative interviewing: The art of hearing data. 2nd ed. Sage: Thousand Oaks (CA); 1995.
Online interviews	Salmons J. Qualitative online interviews. 2nd ed. Sage: London; 2015.
Focus group discussion	Barbour RS, Kitzinger J. Developing focus group research. Politics, theory and practice. 1st ed. Sage: London; 1999.Kruger R, Casey M. Focus groups: A practical guide for applied research. Sage: Thousand Oaks (CA); 2015.

Box 4. Qualitative data analysis.

**Table:** 

	Ethnography	Phenomenology	Grounded theory	Content analysis
Transcripts mainly from	Observations, face-to-face and focus group discussions, field notes.	Face-to-face in- depth Interviews.	Face-to-face in- depth interviews; rarely observations and sometimes focus group discussions.	Face-to-face and online in-depth interviews and focus group discussions; sometimes observations.
Reading, notes and memos	Reading through transcripts, classifying into overarching themes, adding marginal notes, assigning preliminary codes.	Reading through transcripts, adding marginal notes, defining first codes.	Reading through transcripts, writing memos, assigning preliminary codes.	Reading through transcripts, adding marginal notes, assigning preliminary codes.
Describing	Social setting, actors, events.	Personal experience.	Open codes.	Initial codes.
Ordering	Themes, patterns and regularities.	Major and subordinate statements. Units of meaning.	Axial coding. Selective coding.	Descriptive categories and subcategories.
Interpreting	How the culture works.	Development of the essence.	Storyline about social process.	Main categories, sometimes exploratory.
Findings	Narrative offering detailed description of a culture.	Narrative showing the essence of the lived experience.	Description of a theory, often using a visual model.	Narrative summary of main findings.

### What are the general features of an interview?

Interviews involve interactions between the interviewer(s) and the respondent(s) based on interview questions. Individual, or face-to-face, interviews should be distinguished from focus group discussions. The interview questions are written down in an interview guide [7] for individual interviews or a questioning route [8] for focus group discussions, with questions focusing on the phenomenon under study. The sequence of the questions is pre-determined. In individual interviews, the sequence depends on the respondents and how the interviews unfold. During the interview, as the conversation evolves, you go back and forth through the sequence of questions. It should be a dialogue, not a strict question–answer interview. In a focus group discussion, the sequence is intended to facilitate the interaction between the participants, and you might adapt the sequence depending on how their discussion evolves. Working with an interview guide or questioning route enables you to collect information on specific topics from all participants. You are in control in the sense that you give direction to the interview, while the participants are in control of their answers. However, you need to be open-minded to recognize that some relevant topics for participants may not have been covered in your interview guide or questioning route, and need to be added. During the data collection process, you develop the interview guide or questioning route further and revise it based on the analysis.

The interview guide and questioning route might include open and general as well as subordinate or detailed questions, probes and prompts. Probes are exploratory questions, for example, ‘Can you tell me more about this?’ or ‘Then what happened?’ Prompts are words and signs to encourage participants to tell more. Examples of stimulating prompts are eye contact, leaning forward and open body language.

Box 5. Further reading on qualitative analysis.

**Table:** 

Ethnography	• Atkinson P, Coffey A, Delamount S, Lofland J, Lofmand L. Handbook of ethnography. Sage: Thousand Oaks (CA); 2001. • Spradley J. The ethnographic interview. Holt Rinehart & Winston: New York (NY); 1979. • Spradley J. Participant observation. Holt Rinehart & Winston: New York (NY); 1980.
Phenomenology	• Colaizzi PF. Psychological research as the phenomenologist views it. In: Valle R, King M, editors. Essential phenomenological alternative for psychology. New York (NY): Oxford University Press; 1978. p. 41-78. • Smith J.A, Flowers P, Larkin M. Interpretative phenomenological analysis. Theory, method and research. Sage: London; 2010.
Grounded theory	• Charmaz K. Constructing grounded theory. 2nd ed. Sage: Thousand Oaks (CA); 2014. • Corbin J, Strauss A. Basics of qualitative research. Techniques and procedures for developing grounded theory. Sage: Los Angeles (CA); 2008.
Content analysis	• Elo S, Kääriäinen M, Kanste O, Pölkki T, Utriainen K, Kyngäs H. Qualitative Content Analysis: a focus on trustworthiness. Sage Open 2014: 1–10. DOI: 10.1177/2158244014522633. • Elo S. Kyngäs A. The qualitative content analysis process. J Adv Nurs. 2008; 62: 107–115. • Hsieh HF. Shannon SE. Three approaches to qualitative content analysis. Qual Health Res. 2005; 15: 1277–1288.

### What is a face-to-face interview?

A face-to-face interview is an individual interview, that is, a conversation between participant and interviewer. Interviews can focus on past or present situations, and on personal issues. Most qualitative studies start with open interviews to get a broad ‘picture’ of what is going on. You should not provide a great deal of guidance and avoid influencing the answers to fit ‘your’ point of view, as you want to obtain the participant’s own experiences, perceptions, thoughts, and feelings. You should encourage the participants to speak freely. As the interview evolves, your subsequent major and subordinate questions become more focused. A face-to-face or individual interview might last between 30 and 90 min.

Most interviews are semi-structured [3]. To prepare an interview guide to enhance that a set of topics will be covered by every participant, you might use a framework for constructing a semi-structured interview guide [10]: (1) identify the prerequisites to use a semi-structured interview and evaluate if a semi-structured interview is the appropriate data collection method; (2) retrieve and utilize previous knowledge to gain a comprehensive and adequate understanding of the phenomenon under study; (3) formulate a preliminary interview guide by operationalizing the previous knowledge; (4) pilot-test the preliminary interview guide to confirm the coverage and relevance of the content and to identify the need for reformulation of questions; (5) complete the interview guide to collect rich data with a clear and logical guide.

The first few minutes of an interview are decisive. The participant wants to feel at ease before sharing his or her experiences. In a semi-structured interview, you would start with open questions related to the topic, which invite the participant to talk freely. The questions aim to encourage participants to tell their personal experiences, including feelings and emotions and often focus on a particular experience or specific events. As you want to get as much detail as possible, you also ask follow-up questions or encourage telling more details by using probes and prompts or keeping a short period of silence [6]. You first ask what and why questions and then how questions.

You need to be prepared for handling problems you might encounter, such as gaining access, dealing with multiple formal and informal gatekeepers, negotiating space and privacy for recording data, socially desirable answers from participants, reluctance of participants to tell their story, deciding on the appropriate role (emotional involvement), and exiting from fieldwork prematurely.

### What is a focus group discussion and when can I use it?

A focus group discussion is a way to gather together people to discuss a specific topic of interest. The people participating in the focus group discussion share certain characteristics, e.g., professional background, or share similar experiences, e.g., having diabetes. You use their interaction to collect the information you need on a particular topic. To what depth of information the discussion goes depends on the extent to which focus group participants can stimulate each other in discussing and sharing their views and experiences. Focus group participants respond to you and to each other. Focus group discussions are often used to explore patients’ experiences of their condition and interactions with health professionals, to evaluate programmes and treatment, to gain an understanding of health professionals’ roles and identities, to examine the perception of professional education, or to obtain perspectives on primary care issues. A focus group discussion usually lasts 90–120 mins.

You might use guidelines for developing a questioning route [9]: (1) brainstorm about possible topics you want to cover; (2) sequence the questioning: arrange general questions first, and then, more specific questions, and ask positive questions before negative questions; (3) phrase the questions: use open-ended questions, ask participants to think back and reflect on their personal experiences, avoid asking ‘why’ questions, keep questions simple and make your questions sound conversational, be careful about giving examples; (4) estimate the time for each question and consider: the complexity of the question, the category of the question, level of participant’s expertise, the size of the focus group discussion, and the amount of discussion you want related to the question; (5) obtain feedback from others (peers); (6) revise the questions based on the feedback; and (7) test the questions by doing a mock focus group discussion. All questions need to provide an answer to the phenomenon under study.

You need to be prepared to manage difficulties as they arise, for example, dominant participants during the discussion, little or no interaction and discussion between participants, participants who have difficulties sharing their real feelings about sensitive topics with others, and participants who behave differently when they are observed.

### How should I compose a focus group and how many participants are needed?

The purpose of the focus group discussion determines the composition. Smaller groups might be more suitable for complex (and sometimes controversial) topics. Also, smaller focus groups give the participants more time to voice their views and provide more detailed information, while participants in larger focus groups might generate greater variety of information. In composing a smaller or larger focus group, you need to ensure that the participants are likely to have different viewpoints that stimulate the discussion. For example, if you want to discuss the management of obesity in a primary care district, you might want to have a group composed of professionals who work with these patients but also have a variety of backgrounds, e.g. GPs, community nurses, practice nurses in general practice, school nurses, midwives or dieticians.

Focus groups generally consist of 6–12 participants. Careful time management is important, since you have to determine how much time you want to devote to answering each question, and how much time is available for each individual participant. For example, if you have planned a focus group discussion lasting 90 min. with eight participants, you might need 15 min. for the introduction and the concluding summary. This means you have 75 min. for asking questions, and if you have four questions, this allows a total of 18 min. of speaking time for each question. If all eight respondents participate in the discussion, this boils down to about two minutes of speaking time per respondent per question.

### How can I use new media to collect qualitative data?

New media are increasingly used for collecting qualitative data, for example, through online observations, online interviews and focus group discussions, and in analysis of online sources. Data can be collected synchronously or asynchronously, with text messaging, video conferences, video calls or immersive virtual worlds or games, etcetera. Qualitative research moves from ‘virtual’ to ‘digital’. Virtual means those approaches that import traditional data collection methods into the online environment and digital means those approaches take advantage of the unique characteristics and capabilities of the Internet for research [10]. New media can also be applied. See Box 3 for further reading on interview and focus group discussion.

## Analysis

### Can I wait with my analysis until all data have been collected?

You *cannot* wait with the analysis, because an iterative approach and emerging design are at the heart of qualitative research. This involves a process whereby you move back and forth between sampling, data collection and data analysis to accumulate rich data and interesting findings. The principle is that what emerges from data analysis will shape subsequent sampling decisions. Immediately after the very first observation, interview or focus group discussion, you have to start the analysis and prepare your field notes.

### Why is a good transcript so important?

First, transcripts of audiotaped interviews and focus group discussions and your field notes constitute your *major* data sources. Trained and well-instructed transcribers preferably make transcripts. Usually, e.g., in ethnography, phenomenology, grounded theory, and content analysis, data are transcribed verbatim, which means that recordings are fully typed out, and the transcripts are accurate and reflect the interview or focus group discussion experience. Most important aspects of transcribing are the focus on the participants’ words, transcribing all parts of the audiotape, and carefully revisiting the tape and rereading the transcript. In conversation analysis non-verbal actions such as coughing, the lengths of pausing and emphasizing, tone of voice need to be described in detail using a formal transcription system (best known are G. Jefferson’s symbols).

To facilitate analysis, it is essential that you ensure and check that transcripts are accurate and reflect the totality of the interview, including pauses, punctuation and non-verbal data. To be able to make sense of qualitative data, you need to immerse yourself in the data and ‘live’ the data. In this process of incubation, you search the transcripts for meaning and essential patterns, and you try to collect legitimate and insightful findings. You familiarize yourself with the data by reading and rereading transcripts carefully and conscientiously, in search for deeper understanding.

### Are there differences between the analyses in ethnography, phenomenology, grounded theory, and content analysis?

Ethnography, phenomenology, and grounded theory each have different analytical approaches, and you should be aware that each of these approaches has different schools of thought, which may also have integrated the analytical methods from other schools (Box 4). When you opt for a particular approach, it is best to use a handbook describing its analytical methods, as it is better to use one approach consistently than to ‘mix up’ different schools.

In general, qualitative analysis begins with organizing data. Large amounts of data need to be stored in smaller and manageable units, which can be retrieved and reviewed easily. To obtain a sense of the whole, analysis starts with reading and rereading the data, looking at themes, emotions and the unexpected, taking into account the overall picture. You immerse yourself in the data. The most widely used procedure is to develop an inductive coding scheme based on actual data [11]. This is a process of open coding, creating categories and abstraction. In most cases, you do not start with a predefined coding scheme. You describe what is going on in the data. You ask yourself, what is this? What does it stand for? What else is like this? What is this distinct from? Based on this close examination of what emerges from the data you make as many labels as needed. Then, you make a coding sheet, in which you collect the labels and, based on your interpretation, cluster them in preliminary categories. The next step is to order similar or dissimilar categories into broader higher order categories. Each category is named using content-characteristic words. Then, you use abstraction by formulating a general description of the phenomenon under study: subcategories with similar events and information are grouped together as categories and categories are grouped as main categories. During the analysis process, you identify ‘missing analytical information’ and you continue data collection. You reread, recode, re-analyse and re-collect data until your findings provide breadth and depth.

Throughout the qualitative study, you reflect on what you see or do not see in the data. It is common to write ‘analytic memos’ [3], write-ups or mini-analyses about what you think you are learning during the course of your study, from designing to publishing. They can be a few sentences or pages, whatever is needed to reflect upon: open codes, categories, concepts, and patterns that might be emerging in the data. Memos can contain summaries of major findings and comments and reflections on particular aspects.

In ethnography, analysis begins from the moment that the researcher sets foot in the field. The analysis involves continually looking for patterns in the behaviours and thoughts of the participants in everyday life, in order to obtain an understanding of the culture under study. When comparing one pattern with another and analysing many patterns simultaneously, you may use maps, flow charts, organizational charts and matrices to illustrate the comparisons graphically. The outcome of an ethnographic study is a narrative description of a culture.

In phenomenology, analysis aims to describe and interpret the meaning of an experience, often by identifying essential subordinate and major themes. You search for common themes featuring within an interview and across interviews, sometimes involving the study participants or other experts in the analysis process. The outcome of a phenomenological study is a detailed description of themes that capture the essential meaning of a ‘lived’ experience.

Grounded theory generates a theory that explains how a basic social problem that emerged from the data is processed in a social setting. Grounded theory uses the ‘constant comparison’ method, which involves comparing elements that are present in one data source (e.g., an interview) with elements in another source, to identify commonalities. The steps in the analysis are known as open, axial and selective coding. Throughout the analysis, you document your ideas about the data in methodological and theoretical memos. The outcome of a grounded theory study is a theory.

Descriptive generic qualitative research is defined as research designed to produce a low inference description of a phenomenon [12]. Although Sandelowski maintains that all research involves interpretation, she has also suggested that qualitative description attempts to minimize inferences made in order to remain ‘closer’ to the original data [12]. Descriptive generic qualitative research often applies content analysis. Descriptive content analysis studies are not based on a specific qualitative tradition and are varied in their methods of analysis. The analysis of the content aims to identify themes, and patterns within and among these themes. An *inductive* content analysis [11] involves breaking down the data into smaller units, coding and naming the units according to the content they present, and grouping the coded material based on shared concepts. They can be represented by clustering in treelike diagrams. A *deductive* content analysis [11] uses a theory, theoretical framework or conceptual model to analyse the data by operationalizing them in a coding matrix. An *inductive* content analysis might use several techniques from grounded theory, such as open and axial coding and constant comparison. However, note that your findings are merely a summary of categories, not a grounded theory.

Analysis software can support you to manage your data, for example by helping to store, annotate and retrieve texts, to locate words, phrases and segments of data, to name and label, to sort and organize, to identify data units, to prepare diagrams and to extract quotes. Still, as a researcher you would do the analytical work by looking at what is in the data, and making decisions about assigning codes, and identifying categories, concepts and patterns. The computer assisted qualitative data analysis (CAQDAS) website provides support to make informed choices between analytical software and courses: http://www.surrey.ac.uk/sociology/research/researchcentres/caqdas/support/choosing. See Box 5 for further reading on qualitative analysis.

The next and final article in this series, Part 4, will focus on trustworthiness and publishing qualitative research [13].
